# An integrated platform for large-scale data collection and precise perturbation of live *Drosophila* embryos

**DOI:** 10.1038/srep21366

**Published:** 2016-02-11

**Authors:** Thomas J. Levario, Charles Zhao, Tel Rouse, Stanislav Y. Shvartsman, Hang Lu

**Affiliations:** 1 School of Chemical & Biomolecular Engineering, Georgia Institute of Technology, Atlanta, GA, USA 30332; 2 Walter H Coulter Department of Biomedical Engineering, Georgia Institute of Technology and Emory University, Atlanta, GA, USA 30332; 3 Department of Chemical and Biological Engineering and Lewis-Sigler Institute for Integrative Genomics, Princeton University, Princeton, New Jersey, USA 08544

## Abstract

Understanding the fundamental principles governing embryogenesis is a key goal of developmental biology. Direct observation of embryogenesis via *in vivo* live imaging is vital to understanding embryogenesis; yet, tedious sample preparation makes it difficult to acquire large-scale imaging data that is often required to overcome experimental and biological noises for quantitative studies. Furthermore, it is often difficult, and sometimes impossible, to incorporate environmental perturbation for understanding developmental responses to external stimuli. To address this issue, we have developed a method for high-throughput imaging of live embryos, delivering precise environmental perturbations, and unbiased data extraction. This platform includes an optimized microfluidic device specifically for live embryos and also for precise perturbations in the microenvironment of the developing embryos. In addition, we developed software for simple, yet accurate, automated segmentation of fluorescent images, and automated data extraction. Using a quantitative assessment we find that embryos develop normally within the microfluidic device. Finally, we show an application of the high-throughput assay for monitoring developmental responses to external stimuli: anoxia-induced developmental arrest in *Drosophila* embryos. With slight modifications, the method developed in this work can be applied to many other models of development and other stimulus-response behaviors during development.

Embryogenesis is a collection of dynamic processes involving cell division, cell growth and death, collective cell movements, cell shape changes, and gene patterning. *In vivo* live imaging of development allows biologists to directly visualize and understand the fundamental principles governing these highly dynamical processes^1^,^2^,^3^. Recent advances in fluorescence microscopy, in combination with endogenous fluorescent reporters, has made it possible to image whole embryos with single cell resolution throughout embryogenesis^4^,^5^,^6^. However, inherent inter-embryo variability and low-throughput imaging methods make acquiring statistically meaningful data about embryonic development difficult. To address this issue, a combination of high-throughput experimental methods (for acquiring large imaging data sets^7^) and image processing software (for rapid and standardized automated data extraction^8^) can be used to increase the statistical power of data analysis methods^9^.

Previously, we developed a microfluidic device that can array hundreds of embryos for end-on imaging in a matter of minutes^10^,^11^. This method was primarily used with pre-processed, fixed embryo samples to quantitatively define the spatial expression distributions of key dorsal-ventral patterning genes^12^,^13^,^14^,^15^. While we showed that it was possible to use the device with live *Drosophila* embryos, we did not fully optimize the device to culture and image *Drosophila* embryos through gastrulation, as well as for even longer culture and observation applications, potentially with perturbations^4^,^16^,^17^,^18^.

Here, we report an optimized microfluidic device for arraying live *Drosophila* embryos for parallelized imaging of dorsal-ventral development with the ability to subject the embryos to rapid and reliable alterations to microenvironments throughout development (Fig. 1). This allows for otherwise impossible dynamic control of the embryo microenvironment while allowing continuous *in vivo* live imaging of the dorsal-ventral plane in several embryos simultaneously. Furthermore, we have developed an image processing and analysis pipeline for the automated and unbiased measurements of developmental rates on-chip, and we show that embryos develop with similar rates as those found using conventional live imaging techniques. Finally, we use the microfluidic device to investigate the effects of environmental perturbations by controlling oxygen availability to *Drosophila* embryos throughout development to investigate the effects of anoxic conditions on developmental arrest. The microfluidic array was used to create dynamic microenvironments during embryo development and monitor embryo responses *in vivo*. Specifically, we briefly exposed embryos to anoxic microenvironments and analyzed anoxia-induced developmental arrest and recovery.

## Results

### Device optimization for live embryo imaging

For anoxic studies on *Drosophila* development, precise control of the embryo microenvironment and the ability to dynamically change the oxygen availability around developing embryos is necessary. Beginning with the embryo trap array from previous works, it was expected the larger microfluidic device would prove difficult to accurately control the on-chip oxygen concentration, as oxygen would readily diffuse through the gas-permeable polydimethylsiloxane (PDMS) device along the fluid flow path. We constructed COMSOL finite element models to investigate the combined fluid and mass transport in the previous design. From our model, it was discovered that a significant oxygen concentration gradient would exist across a microfluidic array as large as our previously designed embryo trap array^10^ (Fig. 2c,d). It was therefore necessary to optimize our initial design by reducing the residence time of the perfusate in order to ensure minimal variability of microenvironments experienced by embryos in the array.

Furthermore, imaging frequency is a major bottleneck in the throughput of live imaging experiments. Here, we are interested in studying the morphological changes in the dorsal-ventral plane of the embryo at ~80 μm from either the anterior or posterior pole throughout stages 4–6 of development at a temporal resolution of 1 minute. This high imaging rate is necessary to visualize the rapid nuclear cycling dynamics that occurs during stage 4 of embryogenesis. Using these imaging parameters, laser scanning confocal microscopy generally allows imaging of ~20 embryos per experiment. Thus, a device that is as large as the original embryo trap would not have offered any advantages or increases for throughput for live embryo imaging.

**Figure 1:**
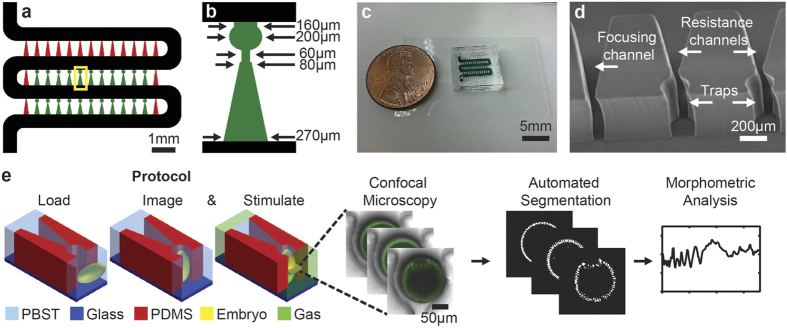
Microfluidic array design and method protocol. (**a**) Overall device layout with main serpentine channel (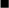), focusing channels (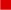), and embryo traps and resistance channels (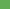). (**b**) Zoom-in of boxed region of (a) with dimensions of the embryo trap and resistance channel. (**c**) Microfluidic array with channels filled with green dye. (**d**) Scanning electron micrograph of trap and focusing channel. (**e**) Method protocol: Load device with live embryos. *In vivo* live imaging of end-on oriented embryos. Delivery of gas-phase stimuli (in this case humidified nitrogen gas), and continue *in vivo* live imaging developmental responses. Confocal microscopy images of live embryo development are then automatically segmented and used for morphometric analysis and data extraction to analyze developmental responses to stimuli.

**Figure 2:**
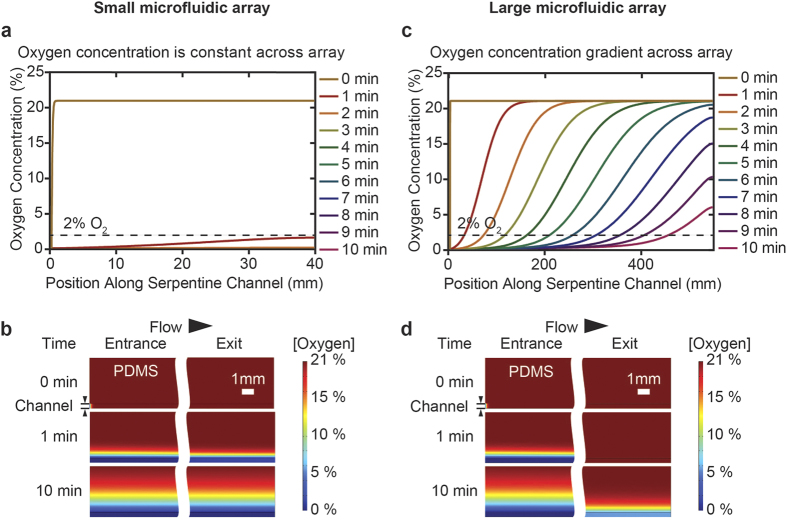
COMSOL simulations of combined fluid and mass transport. (**a**) Line plots depicting oxygen concentration along the serpentine channel as a function of time in a microfluidic array with dimensions equivalent to that described in this paper. After 1 minute of circulating anoxic gas there is <2% oxygen present within the serpentine channel, and <0.1% oxygen after 2 minutes. (**b**) Surface plots depicting oxygen concentration in the serpentine channel and PDMS at the entrance and exit of the small microfluidic array. Constant oxygen concentration in the serpentine channel from entrance to exit of the small microfluidic array. (**c**) Line plots depicting oxygen concentration along the serpentine channel as a function of time in a microfluidic array with dimensions equivalent to that described by our previous large array^10^,^11^. Even after 10 minutes of circulating anoxic gas there is still a significant oxygen concentration gradient along the serpentine channel. (**d**) Surface plots depicting oxygen concentration in the serpentine channel and PDMS at the entrance and exit of the large microfluidic array. Significant oxygen concentration gradient in the serpentine channel from entrance to exit of the large microfluidic array.

For these reasons, the device was miniaturized such that the time-lapse imaging device now contains 22 embryo traps in a 1.7 × 5.5 mm device footprint (Fig. 1a). Twenty-two traps was selected specifically, because array devices such as this have been shown to reach loading efficiencies of >90%; thus, attaining ~90% loading efficiency, we are then able to array our target goal of 20 embryos for each imaging experiment. Modeling results for the small array (Fig. 2a,b) indicate that oxygen can be rapidly removed through the serpentine channel such that <2% oxygen is present throughout the microchannels within 1 minute of switching to humidified nitrogen gas perfusate, and <0.1% oxygen is present after 2 minutes (Fig. 2b). This is significant, because it has been shown that an oxygen concentration of 2% or less is sufficient to elicit cell cycle arrest^19^. In contrast, in the larger array, the convection is not enough to rapidly reduce the oxygen content (Fig. 2c,d).

**Figure 3:**
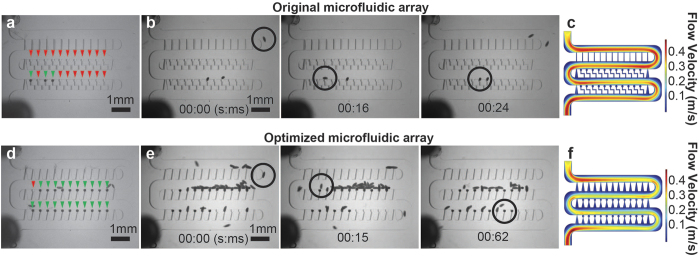
Device loading. (**a**) Typical device loading for microfluidic array employing previously developed embryo trap^10^,^11^. 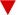 indicate empty traps, 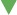 indicate traps with embryos successfully loaded. (**b**) Frames from live embryo loading in (a) showing embryos do not successfully trap in device. ○ is tracking a single embryo through successful trapping. (**c**) COMSOL simulation of the non-optimized microfluidic array exhibiting Dean flow and high flow velocity in the serpentine channel relative to embryo traps and resistance channels. (**d**) Typical device loading for optimized microfluidic array. 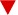 indicate empty traps, 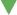 indicate traps with embryos successfully loaded. (**e**) Frames from live embryo loading in (e) showing embryos successfully trap in device. ○ is tracking a single embryo through successful trapping. (**f**) COMSOL simulation of the optimized microfluidic array exhibiting Dean flow and lower flow velocity in the serpentine channel relative to the non-optimized microfluidic array in c).

Scaling the embryo array design from an original size footprint of 50 × 17 mm down to the current 1.7 × 5.5 mm footprint was not a trivial process, because the operational principle of the device relies on the balancing of two perpendicular flow fields throughout the device to efficiently capture embryos. The two flow fields that govern efficient embryo capture are 1) the main flow along the larger serpentine channel, and 2) the secondary flow through the embryo traps arrayed along the serpentine channel. Reducing the number of columns (i.e. traps) along a row in the array decreases the average flow rate through the embryo traps along a given row (Fig. 3c). As a result, a smaller array of the original trap design exhibited an average loading efficiency of 26% (3 experiments with n = 9, 5, and 3 embryos trapped) (Fig. 3a,b). This suggests that the ratio of hydraulic resistances between flow through embryo traps and flow along the serpentine channel are improperly balanced in the smaller array. Embryo-loading videos indicated that Dean flow^20^,^21^,^22^ is achieved in these smaller devices, which successfully placed embryos along the bottom of the serpentine channel near trap entrances; however, the cross-flow through the traps was not strong enough to drive embryos into traps (Fig. 3b,c). To address this issue, we redesigned the microfluidic architecture to restore balance to the flow fields throughout the device and improve embryo trapping.

In order to compensate for the decreased fluid flow through the traps, we aimed to reduce hydraulic resistance of the embryo traps. This was accomplished by first decreasing the length of the resistance channel in the embryo trap unit. However, this did not effectively reduce the hydraulic resistance sufficiently to improve embryo trapping, as average loading efficiency for such design was 64% (3 experiments with n = 17, 14, and 11 embryos trapped) (Supplementary Fig. 1). We further refined the resistance channel geometry such that the resistance channel tapered from 80 μm wide to 270 μm (Fig. 1b). With this design, loading efficiency improved to 91% (3 experiments with n = 21, 20, 19 embryos trapped) (Fig. 3d,e). COMSOL simulations confirmed that the flow velocity in the serpentine channel is significantly decreased relative to flow velocity through traps in this design when compared to the original embryo trap design (Fig. 3f). It should be noted that although sufficiently high flow rates are desired through the embryo traps, very low hydraulic resistances through embryo traps can result in embryos clogging the serpentine channel and prevent loading of additional traps downstream. Thus, a precise ratio of flow rates through the embryo traps and serpentine channel must be achieved to guide the embryos into the traps, but also allow following embryos to pass occupied traps to the next vacant trap downstream.

It is important to note that in addition to balancing the ratio of hydraulic resistances between the embryo traps and main serpentine channel, there is an additional key operational principle at work in the embryo trap array devices - the use of Dean flow. Dean flow is necessary to bring embryos into the proximity of traps, as streamlines create a mixing effect in which Dean forces push embryos from the middle of the serpentine channel to the sidewalls. Without Dean flow, the optimized trap design will not exhibit high loading efficiencies (data not shown). Here, optimal embryo trapping was obtained with ~6 ml min^−1^ volumetric flow rates during embryo loading. At this flow rate, Dean flow begins to have an effect on embryo locations along the width of the serpentine channel.

An additional design parameter is trap geometry. Optimization of this feature required two major changes. The first regarded the overall size of the trap. The trap is a truncated-cylinder of 500 μm tall and 200 μm in diameter. The diameter of the trap was expanded from the original 150 μm in order to reduce pressure exerted from the PDMS trap on the embryo, and improve viability of live embryos on-chip. The second major change regards the trap entrance, which was previously designed to be 90 μm wide. This allowed robust trapping such that fluidic connections could be removed and devices could be easily transported from stereomicroscopes, which are preferred for device loading to higher resolution microscopes such as confocal microscopes. However, although the trap does flex upon slight positive pressure during loading, higher pressures are typically required to direct embryos completely into traps of this shape. Both the added pressure and strain imparted on embryos as they squeeze through the trap entrance can have harmful effects on live embryos.

To reduce the strain imparted on developing embryos during device loading, we optimized the trap entrance such that embryos can enter traps readily at 6 psi, which is an acceptable pressure for handling live organisms^9^,^23^,^24^, and yet still be robustly held in traps for long-term imaging without active circulation of media. To do this, traps with various entrance widths were constructed and tested. We found that trap entrances that approach 200 μm do not robustly hold embryos for end-on imaging; once flow is halted, embryos immediately fall out of the traps into the serpentine. Also, traps with entrances of 170–190 μm wide held embryos well in static flow conditions, but could not robustly handle embryos through issues like transporting devices from low-resolution microscopes to higher resolution microscopes, or accidental bumping of devices. The widest entrance that exhibited robust embryo handling throughout imaging and resisted other complicating factors was 160 μm. Taken together, the optimized device we developed can robustly orient ~20 live embryos for long-term, end-on imaging in less than 3 minutes (Fig. 1).

### Live embryo imaging on-chip shows normal developmental progression

In order to test the compatibility of the device for imaging live *Drosophila* embryos, we first loaded the array and imaged embryo development of Histone-GFP expressing embryos under static conditions in which no active circulation of perfusate was utilized. This is the simplest way to use the device for live imaging. Furthermore, PDMS is gas permeable allowing free diffusion of oxygen from the outside environment toward respiring embryos. Operating with no circulation will allow us to test whether embryos receive sufficient oxygen while developing on-chip, which can be validated by analyzing developmental phenotypes and rates, and comparing with what is known in the literature.

**Figure 4:**
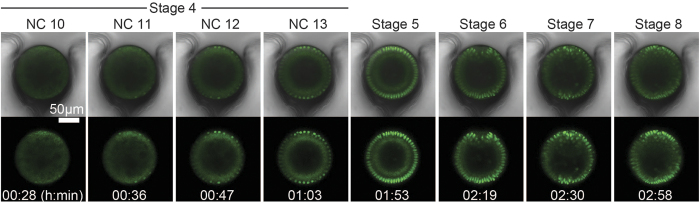
On-chip culture and *in vivo* live imaging of dorsal-ventral development. Each pair of images are single time points from a time-lapse imaging video of a live developing embryo expressing a Histone-GFP transgene within the microfluidic array. The video was 3 hours in total length, and each time-point is stamped with the time that each event occurred. The time is relative to the start time when the confocal microscope began scanning. Top image is a merge of brightfield and Histone-GFP channels, while the bottom image is only the Histone-GFP channel. From left to right: nuclear cycle 10, nuclear cycle 11, nuclear cycle 12, nuclear cycle 13, stage 5, stage 6, stage 7, and stage 8.

Frames from time-lapse imaging of a Histone-GFP expressing embryo are shown in Fig. 4. Typically, embryos are stage 3 or younger when these experiments begin, which is characterized by the absence of nuclei at the periphery of the embryo. Cytoplasmic contractions can be seen during these early stages of development in the GFP channels indicating that synchronous divisions are occurring deep within the yolk beyond visibility. Approximately 25 minutes into imaging, nuclei began to appear at the periphery (Fig. 4), which marks the beginning of stage 4 of embryogenesis. During stage 4, for the next ~45 minutes, the embryo proceeded through 4 synchronous nuclear divisions (Fig. 4) after which the embryo enters stage 5 of development. Stage 5 lasted ~60 minutes in which the syncytial *Drosophila* embryo becomes cellularized to form the blastula (Fig. 4). After cellularization, ventral furrow invagination is initiated during stage 6 (Fig. 4), and invagination completes during stage 7 (Fig. 4). Finally, the invaginated mesoderm collapses and proceeds through the process of mesoderm spreading during stage 8 of development (Fig. 4). It was found that chemically dechorionated His-GFP expressing embryos cultured off-chip proceed through these stages of development with 60% survival rate (n = 126 out of 208 embryos), whereas when cultured on-chip results in 59% survival rate (n = 35 out of 59 embryos). Qualitatively embryos appear to exhibit normal developmental phenotypes within the microfluidic array, and to further confirm this we quantitatively assess on-chip developmental rates, shown in the following section.

### Nuclear cycle kinetics analysis indicates embryos are developing at a normal rate on-chip

With the device optimized for live imaging, we collected live imaging data and compared on-chip development to what was reported in the literature. Previously, Foe and Alberts provided quantitative information about nuclear cycle kinetics in the *Drosophila* syncytial blastoderm^25^. We collected similar data by live imaging on-chip and analyzing the nuclear cycle kinetics during embryonic stages 4 and 5. By doing this, we can quantitatively measure developmental rates on-chip and check if normal development is achieved within the microfluidic device.

**Figure 5:**
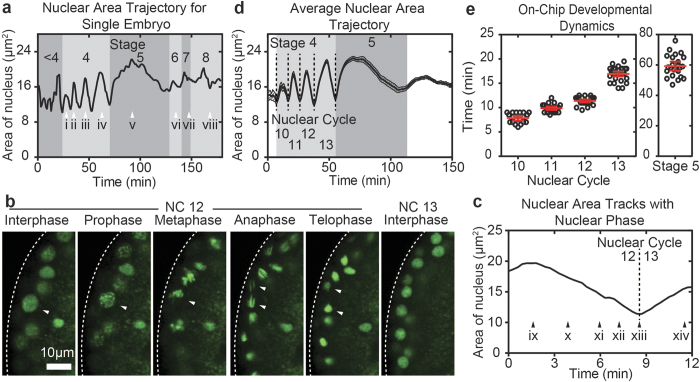
Matlab-based image processing and analysis for quantifying on-chip developmental rates. (**a**) The average area of a nucleus trajectory for a single embryo; the same embryo found in Fig. 4. i–viii indicate the time point at which each image in Fig. 4 was taken. i) Stage 4, nuclear cycle 10. ii) Stage 4, nuclear cycle 11. iii) Stage 4, nuclear cycle 12. iv) Stage 4, nuclear cycle 13. v) Stage 5. vi) Stage 6. vii) Stage 7. viii) Stage 8. (**b**) 63X confocal imaging of Histone-GFP expressing embryo and nuclear cycle phases. indicates the same nuclei proceeding from nuclear cycle 12 interphase to prophase to metaphase to anaphase to telophase. After telophase, the nuclei was lost due to significant z-drift caused by yolk contractions during nuclear cycle 13 interphase. (**c**) Nuclear area trajectory for embryo found in (b). ix-xiv indicate the time point at which each image in (b) was taken. ix) Interphase, nuclear cycle 12. x) Prophase, nuclear cycle 12. xi) Metaphase, nuclear cycle 12. xii) Anaphase, nuclear cycle 12. xiii) Telophase, nuclear cycle 12. xiv) Interphase, nuclear cycle 13. (**d**) Average nuclear area trajectory for embryos developing on-chip exhibits stereotyped oscillations that corresponds with stage 4–5 of development (n = 35 embryos). (**e**) Measured nuclear cycle duration for Histone-GFP expressing embryos developing on-chip as extracted by examining the peak widths found in (d). Average ± S.E.M. duration of nuclear cycle 10, 11, 12, 13, and stage 5 are 7.8 ± 0.2 minutes (n = 27 embryos), 9.9 ± 0.2 minutes (n = 32 embryos), 11.4 ± 0.1 minutes (n = 35 embryos), 16.9 ± 0.2 minutes (n = 35 embryos), and 59.3 ± 1.4 minutes (n = 25 embryos), respectively.

To complement our high-throughput experimental method developed here, we have also developed automated data-analysis approaches. Specifically, we developed an algorithm for automatic identification and segmentation of fluorescent images. In this case of analyzing nuclear cycle kinetics, segmentation is identifying individual nuclei (by Histone-GFP fluorescence) in time-lapse videos. With the nuclei identified, the program then calculates a library of user-defined features that can be used to extract important information about cell cycle kinetics. A feature we found useful is the “average area of a nucleus” or “nuclear area” for short. When plotted as a function of time, the nuclear area exhibits an oscillatory behavior (Fig. 5a). The peaks and valleys correspond to nuclear cycles 10–13 and stage 5, wherein the peaks correspond to interphase and the valleys correspond to the nuclear division phase. This simple measure allows easy and unbiased identification of nuclear cycling kinetics, and provides an assessment for developmental rate on-chip.

In order to understand what phase of the nuclear cycle the quantified “nuclear area” represents, we imaged Histone-GFP expressing embryos using a 63X oil immersion objective and analyzed the videos with the same segmentation algorithm (Fig. 5b). We found that the peak maxima are associated with interphase as nuclei exhibit a round morphology with uniform intensity at these time points (Fig. 5b,c). Nuclear area decreases as nuclei progress to prophase as chromosomes begin to condense, which is identified by nuclei exhibiting a more punctate appearance (Fig. 5b,c). The decrease in nuclear area continues as chromosomes condense further and align at the nuclear division plane during metaphase (Fig. 5b,c). The sister chromatids are segregated during anaphase wherein chromatids can be seen with an elongated morphology (Fig. 5b,c). Finally, the nuclear area goes to minima during telophase as chromosomes are now in a rounded morphology, and still highly condensed prior to chromosome expansion (Fig. 5b,c) that occurs during the next interphase (Fig. 5b,c). Therefore, the minimum to minimum distance in nuclear area trajectories can be used to extract the length of each nuclear cycle during stage 4 of development, wherein specifically these values are measuring the time from one telophase to the next.

Recording large numbers of embryo development time-sequences and averaging feature trajectories allows us to establish how an average embryo develops when cultured in the microfluidic array and compare to the literature. This is helpful when comparing embryo development under different experimental conditions. Because embryos are not perfectly age-synchronized, videos (and therefore feature trajectories) must first be aligned in the developmental time frame. Here, we used nuclear division 12 as the developmental time point with which to align all videos (i.e. the nuclear division between nuclear cycles 12 and 13). Accurate and unbiased alignment of videos in time is made simple, because the code can easily identify nuclear division 12 as a single time-point by looking for local minima in the nuclear area trajectory. Alignment of videos thus allows us to average feature trajectories in order to establish an average embryo developing inside the microfluidic array (Fig. 5d).

The population average (n = 35 embryos from 3 experiments) of the nuclear area trajectory exhibits a stereotyped pattern of 5 peaks that correspond with nuclear cycles 10–14 encompassing stages 4–5 of development. The peak widths are then used to directly extract the duration for each nuclear cycle. The observed duration for nuclear cycles 10, 11, 12, 13 and stage 5 are as follows: 7.8 ± 0.2 minutes (n = 27 embryos), 9.9 ± 0.2 minutes (n = 32 embryos), 11.4 ± 0.1 minutes (n = 35 embryos), 16.9 ± 0.2 minutes (n = 35 embryos), and 59.3 ± 1.4 minutes (n = 25 embryos) (average ± standard error of mean S.E.M.)) (Fig. 5e), which closely matches with what is known to occur for these stages of development when using more conventional imaging methods^25^. Specifically, we see that the duration for each nuclear cycle increases progressively from one cycle to the next starting at cycle 10. Additionally, the relative increases from cycle to cycle are similar to what is expected; i.e. moderate increases in duration from cycle 10 to 11 to 12, a more substantial increase in the duration of cycle 13, and an even more substantial increase in the duration for stage 5. It is evident that the device can effectively array live embryos in order to collect large imaging data sets for statistical analysis of developmental dynamics.

### Effects of anoxia exposure are short-lived and delay overall development

Embryogenesis in humans and flies alike requires sufficient oxygen availability for normal development^26^,^27^,^28^,^29^. Fluctuations in nutrient availability are inescapable, and yet developing embryos must be robust to these changing conditions throughout development. Typically, to directly visualize the effects of oxygen availability on *Drosophila* embryogenesis, experimentalists apply gas with specific oxygen concentrations directly over embryos mounted on a glass slide^19^,^30^,^31^,^32^,^33^. Responses are then monitored with i*n vivo* live imaging with either differential interference contrast imaging or fluorescent reporters^30^,^34^. However, not much work has been done to precisely characterize these responses, which is primarily due to the fact that traditional methods are limited to imaging one or two embryos at a time. This is because embryos must be tediously hand-mounted with embryo glue to prevent embryos from floating away during imaging. Here we apply our method of microfluidic high-throughput live imaging and analysis to quantitatively assess kinetics of anoxia-induced developmental arrest and recovery.

**Figure 6:**
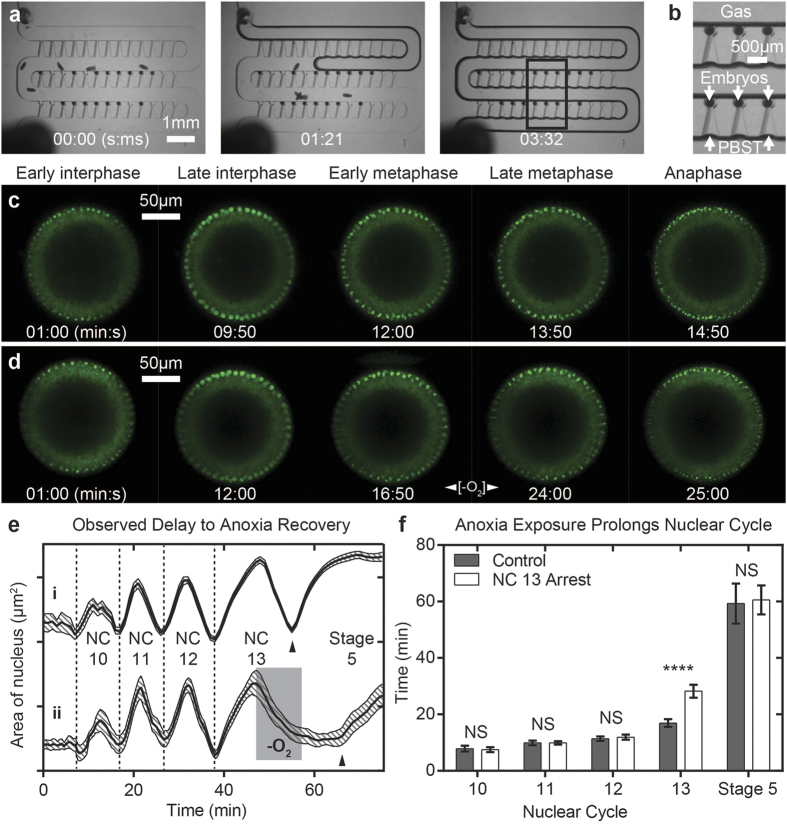
*In vivo* live imaging and quantitative analysis of anoxia-induced developmental arrest on-chip. (**a**) Frames from video showing delivery of humidified nitrogen gas to live embryos on-chip. (**b**) Zoom-in of boxed region in (a) indicating embryos remain oriented for end-on imaging with a reservoir of PBST found in resistance channels that keep embryos from drying out during anoxia exposure. (**c**) Frames from *in vivo* live imaging a Histone-GFP expressing embryo progressing through nuclear cycle 13 (control). (**d**) Frames from *in vivo* live imaging a Histone-GFP expressing embryo progressing through nuclear cycle 13 that exhibits anoxia-induced metaphase arrest. (**e**) Average ± S.E.M. nuclear area trajectory for embryos grown in (i) normoxia (n = 35 embryos), and (ii) experiencing 10 minutes of anoxia during nuclear cycle 13 (n = 14 embryos). Shaded region in (ii) indicates when anoxia was delivered. Black triangle indicates telophase to interphase 14 transition. (**f**) Average ± S.D. durations for nuclear cycles 10, 11, 12, 13, and stage 5 for embryos grown in normoxia (control, n = 35 embryos), and experiencing 10 minutes of anoxia during nuclear cycle 13 (NC 13 arrest, n = 14 embryos). Nuclear cycles 10–12 and stage 5 durations are not significant (NS) while nuclear cycle 13 is statistically different from control (****p < 0.0001. T-test).

In order to investigate anoxia-induced arrest and recovery, we perturbed development by briefly exposing embryos to anoxia while on-chip. To do this, we delivered anoxic pulses of 10 minutes to embryos on-chip by flowing humidified nitrogen gas through the serpentine channel (Fig. 1e). This method allows for rapid changing of the microenvironment as the entire array can be perfused in less than 4 seconds (Fig. 6a). It is possible to do so because the PDMS traps securely hold embryos in place despite active perfusion through the microchannels. Furthermore, the surface tension of PBST is strong enough to resist displacement by the gas phase in the traps and resistance channels, which act as a reservoir of water so that embryos do not dry out during anoxia exposure (Fig. 6b). Finally, this method allows us to directly visualize the dorsal-ventral plane and the morphological responses in the dorsal-ventral plane to anoxia, a feat that to our knowledge has never been accomplished before.

We next analyzed the effects of 10 minute anoxia exposure on nuclear cycle kinetics quantitatively. Embryos that are in nuclear cycle 13 of development when the anoxia exposure occurs exhibit classic signs of anoxia-induced metaphase arrest. During metaphase arrest, nuclei proceed through each phase of the nuclear cycle until metaphase is reached. Under normoxic conditions, nuclei remain in metaphase for ~1–2 minutes (Fig. 6c). However, under anoxic conditions, nuclei exhibit a hypercondensed morphology (Fig. 6d). Embryos remain in this arrested state with hypercondensed nuclei throughout the remainder of the anoxia exposure, and continue to be arrested for several minutes after normoxia is re-established in the device (Fig. 6e). We quantitatively measured that embryos proceeded to anaphase approximately 7.9 ± 0.7 (n = 14 embryos) minutes after embryos were returned to normoxia, which is consistent with what was previously observed^30^,^34^.

Because this method allows us to continuously image embryo development throughout anoxia exposure, we can quantitatively measure developmental rates prior to and after anoxia exposure. By doing this, we find that anoxia-exposed embryos have quantitatively the same nuclear cycle kinetics as control embryos prior to anoxia exposure. This indicates that embryos are viable and developing at normal rates prior to anoxia exposure (Fig. 6f). This is expected, because prior to anoxia exposure, the on-chip conditions are the same as control groups (i.e., embryos are in non-circulating PBST). Embryos that experienced anoxia during nuclear cycle 13, however, were quantitatively measured to have a nuclear cycle 13 duration of 28.0 ± 0.6 minutes (n = 14 embryos) (Fig. 6f). Therefore, 10 minutes of anoxia exposure during nuclear cycle 13 resulted in an increase of ~11.1 ± 2.3 minutes in the duration of nuclear cycle 13. This suggests that *Drosophila* embryos arrest and recover from anoxia-induced developmental arrest with similar kinetics.

**Figure 7:**
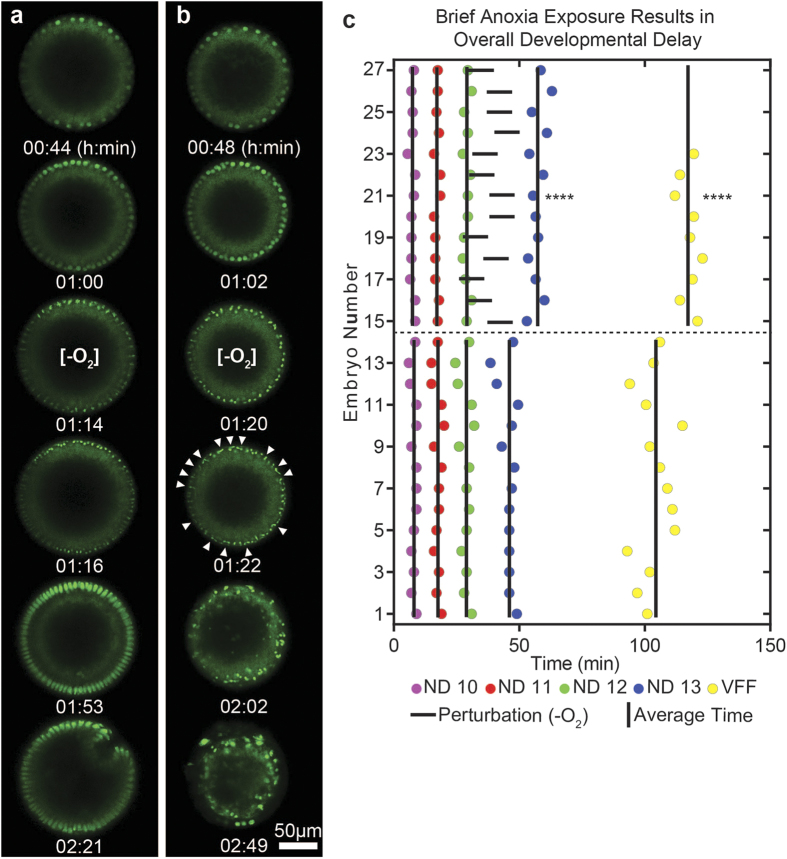
Recovery from anoxia-induced developmental arrest. (**a**) Frames from *in vivo* live imaging a Histone-GFP expressing embryo that successfully recovers from anoxia-induced developmental arrest. From top to bottom: nuclear cycle 12, nuclear cycle 13, nuclear cycle 13 arrest in metaphase, nuclear cycle 13 anaphase-telophase transition, stage 5, and ventral furrow formation. (**b**) Frames from *in vivo* live imaging a Histone-GFP expressing embryo that does not recover from anoxia-induced developmental arrest. From top to bottom: nuclear cycle 12, nuclear cycle 13, nuclear cycle 13 arrest in metaphase, nuclear cycle 13 anaphase-telophase transition (white triangles indicate fused daughter nuclei), nuclear delamination (final two frames). (**c**) Plots showing the timing of milestone events for individual embryos on-chip with population averages for the timing of each event (|). Milestones include nuclear division (ND) 10, 11, 12, and 13, and ventral furrow formation (VFF). Embryos 1–14 are grown entirely in normoxia, and embryos 15–27 are exposed to brief anoxia in nuclear cycle 13 (anoxia indicated by **−**). The timing of nuclear division 13, and ventral furrow formation are statically different from control (****p < 0.0001. T-test).

After anoxia exposure, embryos recover from arrest and continue through the next phases of development, in this case, stage 5 cellularization and stage 6 ventral furrow formation. Previously, it has been shown that embryos exhibit decreased viability when exposed to anoxia prior to cellularization^30^,^35^. We observed the same effect using our method. Embryos were found to successfully recover from anoxia-induced developmental arrest in nuclear cycle 13 ~64% of the time (i.e. 9 out of 14 embryos recover). Successful recovery was taken as the observation of gastrulation (i.e. ventral furrow formation) after arrested development (Fig. 7a). Embryos that did not successfully gastrulate after arrested development exhibited atypical morphology (Fig. 7b). We observed that sister chromatids frequently segregate unsuccessfully from one another after anoxia-induced arrest-the presence of Histone-GFP signal being visibly present between two daughter nuclei that eventually fused together. Many of the nuclei in the field of view appeared to exhibit this same phenotype after anoxia-induced arrest resulting in massive polyploidy and eventually, massive delamination of nuclei from the plasma membrane at the embryo periphery. This suggests that the sensitivity of syncytial embryos to anoxia/hypoxia-induced developmental arrest could be related to massively unsuccessful nuclear divisions prior to cellularization.

Embryos that successfully gastrulate after anoxia-induced arrest exhibit qualitatively normal morphologies in the dorsal-ventral plane (Fig. 7a). Nuclei extend in the apical-basal direction during cellularization and a typical ventral furrow forms during stages 6 and 7 of development. Relative to the start of stage 4, embryos exhibit a statistically significant delay in ventral furrow formation after anoxia-induced arrest when compared to control embryos (Fig. 7c). The delay in ventral furrow formation was measured to be ~14.1 ± 4.2 minutes (n = 9 embryos), which indicates anoxia-induced arrest results in overall developmental delays. This is a phenomenon well-known in the literature. Interestingly, however, the duration of stage 5 in anoxia-exposed embryos is measured to be statistically the same as control embryos indicating that embryos proceed with the same developmental rates immediately after recovery (Fig. 6f). Together, this set of data suggests that the effects of anoxia exposure are immediate, but short-lived.

## Discussion

The combination of endogenous fluorescent reporters and *in vivo* live imaging is a powerful tool for understanding development as it allows biologists to directly visualize dynamic processes in living organisms. Yet, at present it is difficult to uncover the relationship between environmental perturbations and developmental responses *in vivo*, owing to the difficulty of controlling the microenvironment around live specimen. We have shown here a platform that integrates microfluidics, automated image processing, and data extraction for high-throughput studies of normal developmental processes and responses to environmental perturbations. This system is capable of rapid delivery of external stimuli to arrayed, live embryos for continuous *in vivo* live imaging. By using automated image processing and data extraction algorithms, it was possible to quantitatively measure the responses of developing *Drosophila* embryos to varying oxygen concentration early in embryogenesis, for example.

Our system relies on a simple microfluidic design with no moving parts or active valving that securely and robustly arrays live embryos for *in vivo* live imaging; because there is no need for specialized equipment, it is easy to set up and therefore can be adopted by other laboratories. The design of the embryo trap array allows easy removal of embryos and sterilization of the device after each experiment, such that the device can be used multiple times if desired. Furthermore, with slight modifications, this method can be applied to other models of development including, for example, *C. elegans* and zebrafish to facilitate studies of developmental variability and environmental sensing. The generalizability of this method should enable widespread use and rapid adoption across the fields of developmental biology.

In addition to the experiments shown here, this method will be useful for other studies of embryogenesis. For instance, incorporation of the MS2 technology will allow scientists to directly monitor gene expression dynamics in the dorsal-ventral plane in a high-throughput manner^36^,^37^. Moreover, this technology will allow detailed studies of other developmental systems and how these systems respond to environmental perturbation. Our method is applicable to many other applications relevant to teratology, and developmental disorder pathology and treatment. For example, this method is directly applicable to studies interested in less-severe forms of hypoxia^19^,^28^,^30^. In addition to investigating varying degrees of hypoxia, one could also study known teratogens of unknown pathology to investigate the early embryonic responses to teratogen exposure such as alcohol^38^. Furthermore, this method can be used with disease models including, for example, microcephaly^39^, to understand the pathology of early embryonic developmental disorders. Finally, it is also straightforward to apply this method to screening therapeutics aimed at treating teratogen exposure or developmental disorders^40^ in a high-throughput manner and enabling quantitative studies throughout developmental biology.

## Methods

### Device fabrication

Polydimethylsiloxane (PDMS; Dow Corning) microfluidic devices were fabricated using a rapid prototyping technique^41^ (Fig. 1). Briefly, device layouts were designed in AutoCAD and printed onto high resolution transparencies to create photomasks (CAD Art Services; Fig. 1a,b). SU8 2100 photoresist was spin coated onto silicon wafers to a thickness of ~500 μm. The SU8 film was then patterned with a negative relief via standard photolithography and using the previously constructed photomask. The unexposed SU8 is removed via chemical development and the master mold is treated overnight with silane vapor ((tridecafluoro-1,1,2,2-tetrahydrooctyl)-1-trichlorosilane; United Chemical Technologies) to facilitate release of PDMS during replica molding.

PDMS devices are replica molded by first pouring a thin layer of 15:1 PDMS onto the surface of the master mold and allowed to cure for 30 minutes at 75 °C. A second layer of 10:1 PDMS is then poured on top and allowed to cure for an additional 3 hours at 75 °C. The softer layer allows the embryo traps to flex open under slight positive pressure (~6 psi) during embryo loading while the stiffer layer makes the overall device rigid for handling. Access holes are then punched through the PDMS mold to create device inlet and outlet. The PDMS mold is finally plasma bonded to a glass coverslip to create the fully enclosed microfluidic device (Fig. 1c).

### *Drosophila* strains

Histone2AV-GFP (Histone-GFP; Bloomington stock center) expressing flies were used to visualize cell migration and quantify cell cycle kinetics.

### Embryo preparation and device loading

*Drosophila* embryos were prepared for live imaging using standard preparation protocols^25^. Briefly, adult flies were placed over a fresh agar plate for 2 hours at 25 °C for embryo synchronization and collection. Chorion membranes were removed from live embryos by soaking in 2.5% sodium hypochlorite (bleach active ingredient; Clorox) for ~1 minute. Embryos were rinsed with 10 ml of deionized water to remove bleach prior to suspending embryos in 15 ml of PBST, which refers to 0.03% Triton X-100 (Sigma Aldrich) in phosphate-buffered saline solution (VWR). PBST was used for device loading as the surfactant helps prevent embryo aggregation and clumping in the microchannels. For off-chip survival rate experiments, embryos were maintained in PBST in a petri dish for a duration of 3 hours, fixed, imaged, and assessed for survival based on embryo structure and cellular organization as indicated by His-GFP expression. Embryos were identified as dead by a lack of His-GFP expression (indicating an embryo that did not make it to stage 4), or significant polyploidy (indicating an embryo that progressed past stage 3, but experienced nuclear delamination/fusion).

PDMS microfluidic devices are mounted on a dissecting microscope for embryo loading. The dissecting scope is preferred for loading as it offers a wide field of view that allows monitoring for loading errors throughout loading. Devices were primed with PBST to remove air bubbles and coat the PDMS surface with surfactant to help prevent embryo clumping in the microchannels (~5 minutes). Once devices are primed and embryos prepared, embryos were then delivered to the microfluidic device by application of slight positive pressure (~6 psi). After device loading, the device is taken to a confocal microscope for time-lapse confocal microscopy.

### Finite element modeling

COMSOL (COMSOL, Inc.) finite element modeling software was utilized to predict the laminar flow patterns and oxygen mass transport within the microfluidic array. We solved for the 2D steady state solution of the incompressible Navier-Stokes equation. The fluid properties are assumed to be equivalent to those of pure water (i.e. density and viscosity). We assumed no slip boundary conditions for all boundaries except inlet and outlet. The inlet superficial velocity was experimentally measured during loading experiments to be on the order of 0.285 m/s, and set as the inlet boundary condition. The outlet was set to an open boundary condition with 0 N/m^2^ normal stress at the outlet. Temperature was set to 298.15 K.

For mass transport simulation, we constructed a cross section of the microfluidic array that consisted of a top layer of 5 mm thick PDMS, and a bottom layer of 500 μm thick nitrogen gas representing the serpentine channel during anoxia perturbation. The length of the layers were specified as 550 mm and 40 mm to represent the stretched out length of the serpentine channels in our previously designed large array^10^ and the small array described here. We first solved for the 2D steady state solution of the Navier-Stokes equation. The fluid properties are assumed to be equivalent to those of pure nitrogen. We assumed no slip boundary conditions for all boundaries except inlet and outlet. The inlet superficial velocity was experimentally measured during loading experiments to be on the order of 0.285 m/s, and set as the inlet boundary condition. The outlet was set to an open boundary condition with 0 N/m^2^ normal stress at the outlet. Temperature was set to 298.15 K. This solution was then fed into the 2D transient model for combined convection and diffusion mass transport. Diffusivity of oxygen in PDMS was set to 3.55 × 10^−9 ^m^2^/s, while the diffusivity of oxygen in nitrogen was set to 1 × 10^−5 ^m^2^/s. Initial conditions include 21% oxygen concentration throughout the microfluidic cross section. Boundary conditions include: no flux at bottom of serpentine channel (glass slide), 0% oxygen concentration at device inlet (humidified nitrogen gas), 21% oxygen concentration at PDMS surfaces facing the outside environment, and 10:1 oxygen partition coefficient at the nitrogen gas:PDMS interface (top of serpentine channel). Ten minutes of operation with a 1 minute time step were solved for to predict oxygen concentrations during anoxia treatment.

### Time-lapse confocal microscopy

Imaging was performed on a Zeiss LSM 710 confocal microscope with a Zeiss EC Plan-Neofluar 40x/1.30 oil DIC M27 objective. Embryos were imaged ~80 μm from either anterior or posterior pole. Temperature was maintained at 25 °C throughout imaging via an environmental chamber. Embryos remained within the PBST solution within the array throughout imaging. Images were acquired every 60 seconds for a duration of 3 hours in order to encompass the events leading up to and including the first movements of gastrulation (i.e. cellularization and ventral furrow formation).

Higher resolution imaging was utilized to visually confirm that nuclear cycle phase correlates with the measured “average area of nucleus”. Imaging of this type was done utilizing a Zeiss LSM 710 confocal microscope with a Zeiss Plan-Apochromat 63x/1.40 oil DIC M27 objective. Embryos were imaged ~50 μm from either anterior or posterior pole. The temperature was maintained at 2 °C throughout imaging via an environmental chamber. Embryos remained within the PBST solution within the array throughout imaging. Images were acquired every 18 seconds for a duration of 2 hours in order to encompass stage 4 of development.

### On-chip oxygen manipulation

To manipulate the oxygen levels on-chip, first devices were loaded with embryos and set-up on the confocal microscope for time-lapse microscopy. Once mounted onto the microscope, tubing was connected to the device inlet and outlet such that humidified gas from a gas cylinder can be pumped through the device serpentine channel (Figs 1e and 6a,b). This was done by connecting gas cylinders of specific gas concentrations to a bubble stone that would bubble the dry gas through deionized water in order to humidify the gas. This was necessary to greatly reduce or eliminate evaporation of the liquid that remained around embryos in the traps and resistance channels as the gas passed through the serpentine channel. The humidified gas would travel to the microfluidic device via a low gas permeable tubing (i.e. polyethylene tubing) at very ~1–3 ml per minute. The low flow rate allowed the gas phase to displace the liquid within in the serpentine channel, but not within the embryo traps and resistance channels. The gas was allowed to flow through the device for 10 minutes after which the device is flushed with aerated PBST to bring the microenvironment back to normoxia. Imaging continued throughout the experiment and continued for an additional 2 hours after the anoxic exposure.

### Cell cycle kinetics quantification

Cell cycle kinetics were extracted from the time-lapse images of 35 individual embryos by using a custom built Matlab (Mathworks) program. Briefly, the algorithm uses a combination of relative difference filtering and clustering to identify and segment nuclei from Histone-GFP channels. Once segmented, the algorithm calculates user-defined features about the segmented nuclei including, for example, “average area of a nucleus” (referred to as nuclear area from this point forward). Cell cycle timing is then extracted by examining peak widths in the nuclear area feature trajectory.

## Additional Information

**How to cite this article**: Levario, T. J. *et al*. An integrated platform for large-scale data collection and precise perturbation of live *Drosophila* embryos. *Sci. Rep.* **6**, 21366; doi: 10.1038/srep21366 (2016).

## Supplementary Material

Supplementary Information
