# Supplementary material for: An integrated platform for large-scale data collection and precise perturbation of live Drosophila embryos
Source: Sci Rep. 2016 Feb 11;6:21366. doi: 10.1038/srep21366 (PMC4750044; doi:10.1038/srep21366)
Supplement: Supplementary Information [file srep21366-s1.pdf]

An integrated platform for large-scale data collection and precise perturbation of live *Drosophila* embryos

Thomas J. Levario, Charles Zhao, Tel Rouse, Stanislav Y. Shvartsman, and Hang Lu.

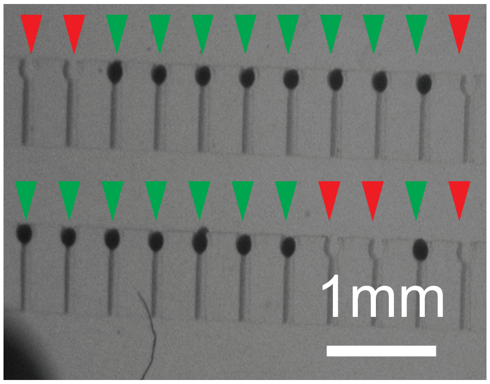

Supplemental Figure 1: Device loading. Typical device loading for microfluidic array employing a straight resistance channel. ▼ indicate empty traps, ▼ indicate traps with embryos successfully loaded.
